# Effects of hypoxic culture conditions on umbilical cord-derived human mesenchymal stem cells

**DOI:** 10.1186/1478-811X-8-18

**Published:** 2010-07-16

**Authors:** Antonina Lavrentieva, Ingrida Majore, Cornelia Kasper, Ralf Hass

**Affiliations:** 1 Institut für Technische Chemie, Leibniz Universität Hannover, Callinstrasse 5, 30167 Hannover, Germany; 2 Laboratory of Biochemistry and Tumor Biology, Department of Obstetrics and Gynecology, Medical University, Hannover, Carl-Neuberg-Straße 1, 30625 Hannover, Germany

## Abstract

Following cultivation of distinct mesenchymal stem cell (MSC) populations derived from human umbilical cord under hypoxic conditions (between 1.5% to 5% oxygen (O_2_)) revealed a 2- to 3-fold reduced oxygen consumption rate as compared to the same cultures at normoxic oxygen levels (21% O_2_). A simultaneous measurement of dissolved oxygen within the culture media from 4 different MSC donors ranged from 15 μmol/L at 1.5% O_2 _to 196 μmol/L at normoxic 21% O_2_. The proliferative capacity of the different hypoxic MSC populations was elevated as compared to the normoxic culture. This effect was paralleled by a significantly reduced cell damage or cell death under hypoxic conditions as evaluated by the cellular release of LDH whereby the measurement of caspase3/7 activity revealed little if any differences in apoptotic cell death between the various cultures. The MSC culture under hypoxic conditions was associated with the induction of hypoxia-inducing factor-alpha (HIF-1α) and an elevated expression of energy metabolism-associated genes including GLUT-1, LDH and PDK1. Concomitantly, a significantly enhanced glucose consumption and a corresponding lactate production could be observed in the hypoxic MSC cultures suggesting an altered metabolism of these human stem cells within the hypoxic environment.

## Introduction

Tissue-derived stem cells including mesenchymal stem cells (MSC) provide enormous potential for appropriate tissue repair and renewal of damaged cells with cellular processes of retrodifferentiation and transdifferentiation extending this spectrum of developmental cellular flexibility and plasticity [1,2]. Thus, human MSC isolated from bone marrow (BM), adipose tissues or other sources are of great interest for tissue engineering and tissue replacement, since these cell populations are characterized by a high proliferative activity, self-renewal capacity, low immunogenicity and the potential to differentiate along the mesenchymal cell lineage to acquire phenotypes of osteoblasts, adipocytes and chondrocytes [3-5]. MSC are already used in clinical trials as cell suspensions [6,7]. For such clinical applications, a large number of cells are required which may still limit the implant preparation in the clinical applications. Another important property is the capability of MSC to survive after implantation when exposed to limited oxygen and nutrient supply due to a lack of vascularization.

Conventional *in vitro *cell cultivation is carried out under ambient oxygen concentration (21% of O_2_) which is also defined as "normoxic". In contrast, *in vivo *MSC usually are not exposed to such a high concentration of oxygen. Depending on the cell type MSC develop in certain environment niches with a low oxygen tension varying between 1% and 7% O_2 _in BM and between 10% and 15% O_2 _in adipose tissue (AT) [8-10]. Common consensus values of 30-50 μM (3% to 5%) of oxygen in tissues are generally accepted whereby the actual O_2 _concentration *in situ *strongly depends on the vascularization of the tissue and its metabolic activity [11]. This suggests hypoxic in vitro conditions for these cells for a treatment under similar conditions resembling their natural physiological environment. Thus, previous work has demonstrated that high concentrations of oxygen can cause oxidative stress via production of reactive oxygen species (ROS) - free radicals that can damage lipids, proteins and DNA, altering cell metabolism [12]. Therefore, moderate hypoxia may lower the intracellular ROS generation and accumulation and may thereby increase the metabolic efficiency [13]. In this context, it is interesting to note that preculturing MSCs under hypoxic conditions before transplantation improves their tissue regenerative potential [14]. Thus, optimal oxygen concentrations must be assigned in accordance with physiological niches and the type of cultivated stem cells, e.g. MSC derived either from bone marrow, peripheral blood, adipose tissue, placenta tissue or from umbilical cord. Under such circumstances, noninvasive optical on-line measurements of dissolved oxygen in the culture medium are very helpful in monitoring and control of cell incubation under conditions of variable oxygen concentration [15].

Birth-associated tissues including amnion, placenta, cord blood and the umbilical cord (UC) represent a very promising embryonic/fetal source of MSC populations and may provide a broader spectrum of cellular flexibility as compared to MSC obtained from adult tissues (bone marrow, adipose tissue). Thus, UC-derived MSC have a short doubling time, their harvest is not ethically restricted and there are no medico-legal limitations in their application [16,17]. Oxygen tension within the mammalian female reproductive tract was shown to be low, about 1.5% to 8% and lasts throughout the fetal development with dissolved oxygen in the fetal circulation rarely exceeding 5% [18,19]. Moreover, the UC with its blood vessels - two arteries and one vein - is lacking capillaries or lymphatic channels providing conditions that UC cells could also develop *in situ *in a hypoxic atmosphere. However, little is known about the effect of hypoxia on UC-derived MSC. Thus, the metabolic potential and the proliferative capacity of these UC-derived MSC at low oxygen tension has not been determined to date. In this study we therefore tested the effects of hypoxic conditions (1.5%, 2.5% and 5% O_2_) on a cultivation of 4 different individual UC-derived MSC populations to examine the proliferative and metabolic activities in these human stem cells.

## Methods

### Cell culture

Human MSCs were isolated from UCs of 4 different term-deliveries (38-40 weeks) by Cesarean section patients after obtaining informed written consent, respectively, as approved by the Institutional Review Board, project #3037 on 17^th ^June, 2006 and in an extended permission #443 on 26^th ^February, 2009. Recently, the isolated populations have been extensively characterized as mesenchymal stem cells by surface marker analysis and functional properties [20]. Moreover, the MSC were expanded and cryopreserved until the start of the experiment as described [20]. After thawing, the cells were expanded over two passages. At about 80% of confluency the MSCs were harvested by accutase treatment (PAA Laboratories GmbH, Pasching, Austria) and plated at a density of 3000 cells/cm^2 ^in 25 cm^2 ^cell culture flasks (Corning, CellBind Surface, Germany) and in 6-well plates (Sarstedt, Germany), respectively. Experiments were performed with cells of passages 3 to 7. Cells were cultivated in αMEM containing 1 g/l glucose (Biochrom, Germany), 2 mM L-glutamine (PAA Laboratories GmbH), 10% human serum (provided by the Division of Transfusion Medicine, Medical University Hannover, Germany) and 50 μg/ml Gentamicin (PAA Laboratories GmbH) in a humidified atmosphere containing 5% CO_2 _and 21% O_2 _at 37°C (Incubators: Thermo scientific, Germany). Twenty four hours after seeding, all non-adherent cells were removed by media changes and for the following 72 h the MSCs were incubated at various oxygen concentrations (1.5%, 2.5%, 5% or 21%), respectively.

### Cell number, apoptosis and necrosis

At the end of cultivation, cells were washed with PBS, detached by accutase treatment (PAA Laboratories GmbH), sedimented by centrifugation for 5 min at 200 × g and counted using a haemocytometer following resuspension in 1 ml culture medium. Cell viability was determined by trypan blue exclusion (n = 4). Occurance of apoptosis was measured with ApoOne^® ^Homogeneous Caspase-3/7 Assay (Promega, UK) by the amount of the fluorescent product Rhodamine 110 (Ex_355_/Em_460_) cleaved by caspase-3/7 from the non-fluorescent substrate Z-DEVD-R110 after cell lysis following 6 h incubation at 37°C. Cell damage or cell necrosis was evaluated by measuring lactate dehydrogenase (LDH) activity in the cell culture supernatant (30 min incubation time, 25°C) using the CytoTox-ONE™ Assay (Promega, UK), by the amount of enzymatically reduced resorufin by its fluorescence intensity (Ex_355_/Em_460_) according to manufacturer's instructions. All fluorescence measurements were performed using the Fluoroscan Ascent microplate reader (Thermo Scientific, Germany).

Normoxic (21% O_2_) cultures of the same passage were used as control cell population and the results of each oxygen concentration were presented as percentage of change to the normoxic controls.

### O_2 _and pH measurements

Dissolved oxygen and pH values in the cell culture supernatant were recorded online in 25 cm^2 ^cell culture flasks (Corning, CellBind Surface, Germany) every 10-20 minutes by using a SFR-Shake Flask Reader (Presens GmbH, Regensburg, Germany) with optical sensors, integrated and precalibrated by Presens GmbH. These measurements are based on the luminescence lifetime of the sensor dye, which depends on the oxygen partial pressure and the pH of the sample, respectively. The luminescence lifetime was detected non-invasively through the transparent flask bottom and represented equivalents of oxygen and pH values according to the company's software (Presens, Germany). Culture flasks with the same amount of medium (6 ml) without cells were used as a control. Oxygen consumption was calculated as difference of dissolved oxygen concentration in medium with and without cells divided by the number of living cells.

### Immunoblot analysis

The isolated MSC cultures following incubation at different oxygen levels (1.5% to 21%) were washed three times in ice-cold PBS and immediately lysed in a buffer containing 10 mM Tris-HCl (pH 7.6), 140 mM NaCl, 10 mM EDTA, 1% (v/v) NP-40 with the addition of 10 μg/ml aprotinin, 10 μg/ml leupeptin, and 1 mM phenylmethylsulfonylfluoride (PMSF) (all from Sigma). Protein concentration was adjusted using the colorimetric BCA-assay (Perbio Science Deutschland, Bonn, Germany), subjected to SDS-polyacrylamide gel electrophoresis and transferred to a PVDF membrane (Millipore GmbH, Schwalbach, Germany). The membranes were blocked with PBS containing 5% FCS and 0.05% Tween-20 (PBS/Tween). After washing four times with PBS/Tween, the membranes were incubated with the primary monoclonal antibodies (anti-HIF-1α (Biomol GmbH, Hamburg, Germany) and anti-ß-actin, clone AC-15 (Sigma, Saint Louis, Missouri, USA)) for 2 h/37°C, washed four times with PBS/Tween and incubated with the appropriate horseraddish peroxidase-conjugated secondary antibody (all from Santa Cruz Biotechnology, Santa Cruz, CA) for 1 h/37°C. The membranes were washed with PBS/Tween and visualized by autoradiography using the ECL-detection kit (GE Healthcare, München, Germany).

### Real-Time RT-PCR (HIF Target genes Analysis)

Total RNA from the cells incubated at different oxygen conditions was isolated by using RNeasy Mini Plus Kit (Qiagen, Germany) according to the manufacturer's instructions and the RNA concentration was measured with a Nanodrop 1000 Spectrophotometer (Thermo Scientific, Germany). Thereafter, 1 μg of RNA was transcribed into cDNA using Reverse Transcriptase (Promega , UK) and a mixture of oligo(dT) primers according to the manufacturer's instructions. Primers for glucose transporter-1 (GLUT-1), lactate dehydrogenase A (LDHA), glucose-6-phosphate dehydrogenase (G6PD), pyruvate dehydrogenase kinase-1 (PDK-1) and hypoxanthine phosphoribosyltransferase-1 (HPRT1) genes were designed using OligoPerfect™ Designer Software (Invitrogen). Quantitative RT-PCR was performed using IQ™SYBR^®^Green Supermix and IQTM5 real-time PCR Detection System (Bio-Rad, USA). HPRT1 gene was used as internal control and non-template control was used as negative control. The dissociation curves were run for all completed SYBR Green reactions to rule out non-specific amplifications and primer-dimers. Data were analyzed using the comparative Ct (ΔΔCT) method. For each sample triplicate measurements were performed.

### Glucose and L-glutamine consumption, lactate and glutamate production (metabolic analysis)

At the end of each cultivation concentrations of glucose and lactate were measured in the medium using an YSI 2700 SELECT analyzer (Yellow Springs, USA). L-glutamine and l-glutamate concentrations were determined using a gradient HPLC (column: Waters Resolve C18, 5 μm, 3.9 × 150 mm, 30°C, flow: 1 ml/min) with Fluorescence Detector RF-10AXL (Shimadzu, Japan).

Specific metabolite consumption rates (*qmet*) were calculated using the following equation:

$$qmet=\frac{\mu }{Cx(0)}\times \frac{C_{met}(t)-C_{met}(0)}{e^{\mu t}-1}$$

with

$$\mu =\frac{\ln\left[Cx(t)/Cx(0)\right]}{\Delta t}$$

whereby *Cx(0) *and *Cx(t) *represent the cell numbers and *Cmet(0) *and *Cmet(t) *the amount of metabolite at the start (0) and the end (t) of the exponential growth phase, respectively, *t *the time (h) and *μ *the specific growth rate (h^-1^).

Lactate to glucose yields were calculated as

$$Y_{lac/glc}=\frac{q_{lac}}{q_{glc}},$$

where *q*_*lac *_and *q*_*glc *_are specific lactate production and glucose consumption rates during the same time interval, respectively.

### Statistical analysis

All data are represented as mean ± SD for triplicate measurements for each sample. Statistical significance was assessed with the one way ANOVA and t-test at P < 0.05.

## Results

### On-line measurements of dissolved oxygen and pH values of the medium

**Figure 1:**
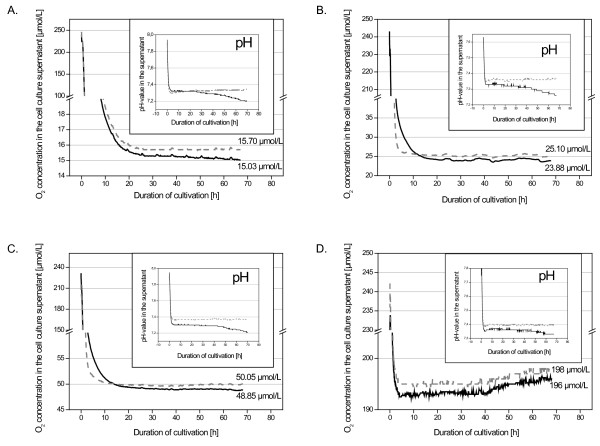
**Results of online-measurements of the dissolved oxygen concentrations and the pH in the cell culture supernatant at 1.5% (A), 2.5% (B), 5% (C) and 21% (D)**. The oxygen concentrations and the appropriate pH were subsequently detected for 70 h in the 4 different cell cultures whereby an average representative kinetic of two MSC donors is demonstrated for each O_2 _concentration. The dissolved oxygen concentration and the corresponding pH values are demonstrated for the cell culture (black solid line) and for a parallel medium control without cells (grey dashed line).

**Figure 2:**
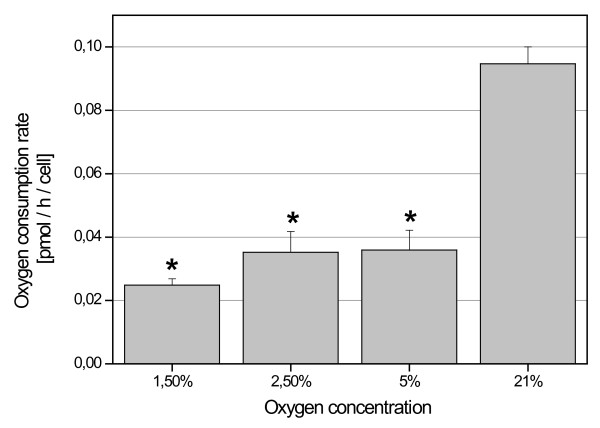
**Rates of oxygen consumption by UC-derived MSC from all donors (n = 4) at 1.5%, 2.5%, 5% and 21% O**_**2**_. Asterisks indicate statistically significant differences in comparison to the normoxic (21% O_2_) control (* p < 0.001).

Twenty-four hours after seeding, the cell culture medium was changed and cells were placed on the SFR-Shake Flask Reader in an incubator with reduced oxygen concentration. For three days, dissolved oxygen concentrations in the medium and pH values were measured and recorded on-line every 10 to 20 minutes (Fig. 1). The measurements showed even at 1.5% O_2 _only a marginal reduction of the available oxygen levels as compared to the appropriate cell-free medium indicating a faster gas diffusion into the medium than the rate of cellular consumption (Fig. 1). Thus, at the end of the cultivations (80% confluency) with 1.5% O_2_, the concentration of oxygen in the cell culture supernatant was 15.03 μmol/l as compared to 15.7 μmol/l in the appropriate medium control (control) (Fig. 1A). Likewise, 2.5% O_2 _incubation revealed 23.88 μmol/l of oxygen in the culture supernatant versus 25.10 μmol/l in the control (Fig. 1B), 5% O_2 _resulted in 48.85 μmol/l versus 50.05 μmol/l in the control (Fig. 1C), and normoxic conditions at 21% O_2 _exhibited 196 μmol/l in the cell culture as compared to 198 μmol/l in the cell-free control medium (Fig. 1D). Accordingly, the calculated oxygen consumption at the end of cultivation (80% confluency) was 0.024 ± 0.002 pmol/h/cell in 1.5% O_2_, 0.035 ± 0.006 pmol/h/cell in 2.5% O_2_, 0.036 ± 0.006 pmol/h/cell in 5% O_2_, and 0.095 ± 0.005 pmol/h/cellin 21% O_2 _(Fig. 2).

Moreover, the pH values in the cell culture supernatant progressively decreased during cultivation time in parallel to an increasing cell number and reached a difference of about 0.15 pH values compared to control medium at the end of the cultivation (80% confluency) (Fig. 1A).

### Cell proliferation, apoptosis and necrosis

**Figure 3:**
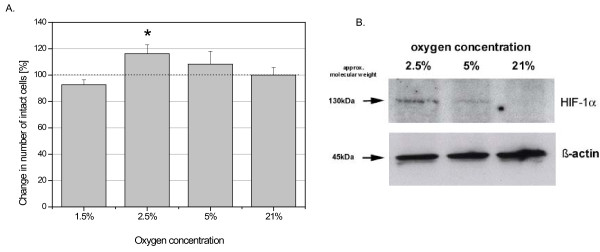
**A. The effect of hypoxia on the cell growth of the UC-derived stem cells from all donors (n = 4)**. Data represent the mean ± SD (*p < 0.05) B. Western Blot of HIF-1α protein expression at different O_2 _concentrations following a 72 h incubation of the MSC populations. The expression of β-actin served as a loading control.

Whereas other studies also use 6 h, 24 h and 48 h of hypoxic incubation, UC-derived MSC were exposed to various concentrations of oxygen for 72 h and the proliferation, apoptosis and cell damage/necrosis in the 4 different UC cell populations was investigated. Cell growth analysis revealed a marked increase in cell proliferation at 2.5% O_2 _as compared to normoxic 21% O_2 _(Fig. 3A). The hypoxic conditions were evaluated by HIF-1α expression and Western blot analysis revealed significant protein levels of HIF-1α in the hypoxic MSC cultures at 2,5% and 5% O_2 _in contrast to little if any detectable HIF-1α protein in the normoxic (21% O_2_) MSC population (Fig. 3B). The unaltered expression of β-actin was used as a control (Fig. 3B).

**Figure 4:**
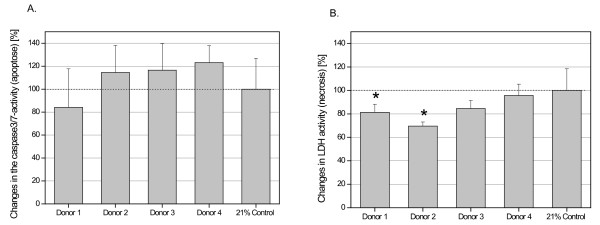
**The effect of hypoxia (1.5% O**_**2**_) on apoptosis was tested by the measurement of caspase 3/7 activity (A) and cell damage or necrosis was tested by the measurement of LDH activity in the cell culture supernatant (B) of the 4 different donors of UC-derived stem cells. All measurements were normalized to 10,000 cells whereby the values of all measurements were calculated compared to the normoxic control conditions (21% O_2_) at 100%.. Data represent the mean ± SD for three independent measurements of each donor.

More detailed analysis of the hypoxic (1.5% O_2_) MSC cultures revealed little if any increase in apoptosis in cell preparations from all four donors (Fig. 4A). In contrast, a markedly decreased cell damage or necrosis in all MSC populations became detectable under hypoxic conditions as evaluated by a significantly reduced LDH release in two of the four MSC donors (Fig. 4B).

### Glucose metabolism-associated gene regulation

**Figure 5:**
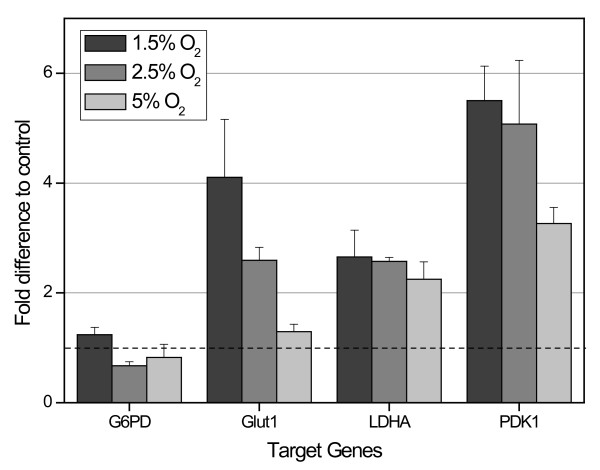
**The effect of hypoxia on glucose metabolism-associated gene expression which may also represent HIF-1α target genes**. Gene expression was detected by Real-Time RT-PCR using primers for glucose-6-phosphate dehydrogenase (G6PD), glucose transporter-1 (GLUT-1), lactate dehydrogenase A (LDHA), pyruvate dehydrogenase kinase-1 (PDK-1) with hypoxanthine phosphoribosyltransferase-1 expression as an internal control. The data represent the expression levels in the UC-derived MSC populations at 1.5% O_2 _(black bars), 2.5% O_2 _(dark grey bars) and 5% O_2 _(light grey bars) and compared to the steady state expression levels of normoxic control cultures at 21% O_2 _(dashed line). Data represent the mean ± SD of three independent experiments.

We also analysed the regulation of some energy metabolism pathway-associated factors including *GLUT-1 *(glucose transport into the cell)*, LDHA *(glycolysis)*, G6PD *(pentose phosphate pathway)*, PDK-1 *(suppression of the oxidative phosphorylation) which also represent some targets of the transcription factor HIF-1α. Quantitative RT-PCT analysis revealed a significant upregulation of *GLUT-1*, *LDHA *and *PDK-1 *in 1.5% O_2_, 2.5% O_2 _and 5% O_2 _as compared to normoxic cultivated (21% O_2_) control cells (Fig. 5). In contrast, no upregulation of *G6PD *in hypoxic conditions was detectable (Fig. 5).

### Metabolic analysis

**Figure 6:**
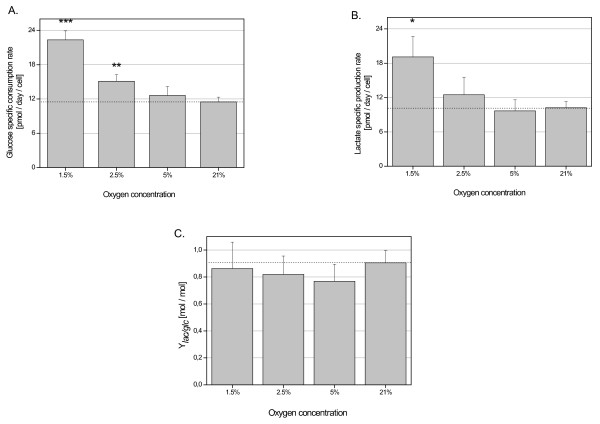
**The effect of hypoxia on the glucose consumption (A), lactate production (B) and yield lactate/glucose (Ylac/glc) (C) of the UC-derived stem cells in all donors**. Data are the means ± SD for triplicate measurements for each donor, four donors per each oxygen concentration. (**p < 0.005, ***p < 0.001, *p < 0.05).

MSC cultured at 1.5% O_2 _consumed significantly more glucose (22.35 ± 1.56 pmol/day/cell) (Fig. 6A) and produced significantly more lactate (19.11 ± 3.58 pmol/day/cell) when compared to normoxic controls (12.00 ± 1.93 and 11. 44 ± 2.93 respectively) (Fig. 6B). At 2.5% glucose consumption and lactate production rates were lower than at 1.5% O_2 _(15.10 ± 1.39 and 12.48 ± 3.08 pmol/day/cell respectively), but still higher than in nomoxic controls (Fig. 6 A, B). At 5% O_2_, there were no differences in glucose uptake and lactate production when compared to 21% O_2_. The calculated lactate/glucose molar ratio was nearly the same at all oxygen concentrations (0.7 - 1.0 mol/mol) (Fig. 6C).

**Figure 7:**
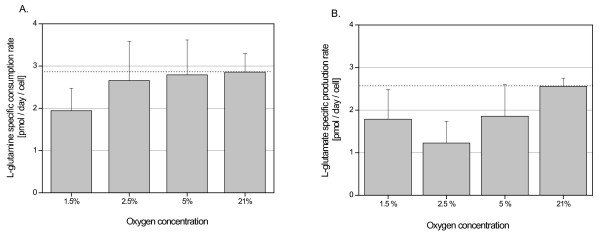
**The effect of hypoxia on the glutamine consumption (A) and glutamate production (B) of the UC-derived stem cells**. Data are the means ± SD for triplicate measurements for each donor, four donors per each oxygen concentration.

Consumption of glutamine was lower at 1.5% O_2 _(1.94 ± 0.53 pmol/day/cell) and at 2.5% O_2 _(2.65 ± 0.95 pmol/day/cell) with no detectable difference at 5% O_2 _(2.79 ± 0.72 pmol/day/cell) when compared to 21% O_2 _controls (2.82 ± 1.37 pmol/day/cell) (Fig. 7A). Glutamate production was lower at 1.5%, 2.5% and 5% O_2 _when compared to 21% normoxic control (Fig. 7B).

## Discussion

Cultivation of MSCs under hypoxic conditions mimic the natural microenvironment of these cells represents an important prerequisite to study cell proliferation, differentiation, senescence, metabolic balance and other physiological processes [14]. Thus, a variety of studies for *in vitro *cell cultivation and subsequent clinical applications suggested the MSC culture at hypoxic (1% to 10% O_2_) rather than normoxic (21% O_2_) conditions [21,22]. Moreover, implanted MSCs in clinical applications without well-developed blood vessels would suffer from limited nutrient and oxygen supply which requires more knowledge about the ability of the cells to survive and adapt to the altered microenvironment.

The results from the present study reveal that UC-derived mesenchymal cells adapt their oxygen consumption and the accompanying energy metabolism according to the appropriate available oxygen concentrations. Thus, oxygen consumption rates of MSC under hypoxic conditions were about 3 times lower as compared to a normoxic atmosphere. Similar data have been reported in hypoxic primary human fibroblasts and the online measurements of the oxygen consumption rate at normoxic conditions stays in the nanomolar range which was in agreement with a variety of other studies [23-26]. Significant up-regulation of the *PDK1 *gene expression confirms previous findings, demonstrated that decreased cell respiration under hypoxic conditions is a result of downregulation of the mitochondrial oxygen consumption [24]. Upregulated PDK1 suppresses the utilization of pyruvate as a fuel for the Krebs cycle. This mechanism is used by the cell to maintain the intracellular oxygen concentration, i.e. to keep homeostasis.

Cultivation of UC-derived human MSC at 1.5% O_2 _revealed little if any increase in apoptosis. Moreover, in 2.5% O_2 _cells demonstrated an increased proliferative capacity. Similar data were obtained in bone marrow-derived MSC [14,27]. Moreover, the level of cell damage and/or necrosis under 1.5% hypoxia was significantly lower than in the normoxic control cell culture suggesting an adaptation to the energy requirements during hypoxia. This reduced concentration of oxygen in the hypoxic environment can contribute to a reduced production and availability of reactive oxygen species which are mainly responsible for the enhancement of cell damage [28,29]. The energy metabolism is mainly represented by glucose and glutamine, two important molecular carbon and nutrient sources. The analysis of metabolic activities of UC-derived MSC in our study were in agreement with previously described increases in glucose consumption and lactate production at low oxygen tension as a consequence of switching cell metabolism from oxidative phosphorylation to anaerobic glycolysis as well as an up-regulation of the glucose transport into the cells [27,30]. The yield of lactate production from glucose, however, was significantly lower in UC-derived MSC than it has been reported in bone marrow- and adipose tissue-derived MSC for both, hypoxic and normoxic conditions [30-33]. One possible explanation could be metabolic modifications within the mesenchymal stem cell populations originating from embryonic/fetal sources like umbilical cord as compared to adult bone marrow or adipose tissue. Indeed, recent work substantiates this hypothesis demonstrating alterations in the capacity of UC-derived MSC to differentiate along the adipogenic, chondrogenic and osteogenic pathway as compared to MSC obtained from adult adipose tissue [34]. This is furthermore supported by the suggestions of different MSC subpopulations exhibiting different levels of proliferative capacity and subsequent aging [20,35]. Likewise, hypoxia-induced MSC from different sources may also display different functional characteristics. Thus, a hypoxic environment can modulate the autocrine or paracrine activity of a variety of cytokines and growth factors in bone marrow-derived MSC [36], whereas in cord blood-derived MSC, two subpopulations were identified displaying low and high aldehyde dehydrogenase (ALDH) activity and significant differences in the proliferative capacity and the ability to differentiate [37].

Together, these findings demonstrated that UC-derived human MSC adapt the energy consumption and metabolism according to an appropriate hypoxic environment. Interestingly, distinct hypoxic conditions contribute to enhanced growth of UC-derived MSC in parallel to reduced cellular damage. Moreover, human mesenchymal stem cells obtained from the umbilical cord displayed metabolic differences during the adaptation to a hypoxic environment when compared to MSC derived from other tissues indicating tissue-originating variations in the functional properties of these different stem cell (sub)populations.

## Competing interests

The authors declare that they have no competing interests.

## Authors' contributions

AL was involved in MSC isolation and primary culture from different umbilical cords and the RT-PCR measurements. IM contributed to the oxygen and enzyme measurements. CK designed the study and drafted the manuscript. RH provided the ethic vote and patient's written consent for the umbilical cords, contributed to the hypoxic culture of UC-MSC, performed the HIF-1α Western Blots and finalized the manuscript. All authors have read and approved the final manuscript.
